# Deficient Recurrent Cortical Processing in Congenital Deafness

**DOI:** 10.3389/fnsys.2022.806142

**Published:** 2022-02-25

**Authors:** Prasandhya Astagiri Yusuf, Aly Lamuri, Peter Hubka, Jochen Tillein, Martin Vinck, Andrej Kral

**Affiliations:** ^1^Department of Medical Physics/Medical Technology IMERI, Faculty of Medicine, University of Indonesia, Jakarta, Indonesia; ^2^Institute of AudioNeuroTechnology and Department of Experimental Otology of the ENT Clinics, Hannover Medical School, Hanover, Germany; ^3^MEDEL Comp., Starnberg, Germany; ^4^Ernst Strüngmann Institut for Neuroscience in Cooperation with Max Planck Society, Frankfurt am Main, Germany; ^5^Donders Centre for Neuroscience, Department of Neuroinformatics, Radboud University Nijmegen, Nijmegen, Netherlands; ^6^Department of Biomedical Sciences, School of Medicine and Health Sciences, Macquarie University, Sydney, NSW, Australia

**Keywords:** spike-field coherence, functional connectivity, congenital deafness, auditory function, electrical recording, cortical column

## Abstract

The influence of sensory experience on cortical feedforward and feedback interactions has rarely been studied in the auditory cortex. Previous work has documented a dystrophic effect of deafness in deep cortical layers, and a reduction of interareal couplings between primary and secondary auditory areas in congenital deafness which was particularly pronounced in the top-down direction (from the secondary to the primary area). In the present study, we directly quantified the functional interaction between superficial (supragranular, I to III) and deep (infragranular, V and VI) layers of feline’s primary auditory cortex A1, and also between superficial/deep layers of A1 and a secondary auditory cortex, namely the posterior auditory field (PAF). We compared adult hearing cats under acoustic stimulation and cochlear implant (CI) stimulation to adult congenitally deaf cats (CDC) under CI stimulation. Neuronal activity was recorded from auditory fields A1 and PAF simultaneously with two NeuroNexus electrode arrays. We quantified the spike field coherence (i.e., the statistical dependence of spike trains at one electrode with local field potentials on another electrode) using pairwise phase consistency (PPC). Both the magnitude as well as the preferred phase of synchronization was analyzed. The magnitude of PPC was significantly smaller in CDCs than in controls. Furthermore, controls showed no significant difference between the preferred phase of synchronization between supragranular and infragranular layers, both in acoustic and electric stimulation. In CDCs, however, there was a large difference in the preferred phase between supragranular and infragranular layers. These results demonstrate a loss of synchrony and for the first time directly document a functional decoupling of the interaction between supragranular and infragranular layers of the primary auditory cortex in congenital deafness. Since these are key for the influence of top-down to bottom-up computations, the results suggest a loss of recurrent cortical processing in congenital deafness and explain the outcomes of previous studies by deficits in intracolumnar microcircuitry.

## Introduction

Deafness is the most frequent sensory disorder (World Health Organization [WHO], 2021). Congenital deafness deprives the child of acoustic input during a developmental period that requires experience to acquire spoken language (Kral and O’Donoghue, 2010). Deafness thus has a serious impact on the development of the child. Cochlear implants effectively compensate for the sensory deficits and allow language development if implanted during the first year of life. However, late implantations are not very successful. While late-implanted prelingually deaf adults can hear with the cochlear implant, they have difficulties discriminating and identifying speech and complex acoustic stimuli. This results in a critical period in managing congenital hearing loss (Kral et al., 2019). What makes the earliest implanted congenitally deaf children star performers, but the late implanted poorest performers? How do cortical circuits in deafness differ from their hearing counterparts? What are the distinctive differences in processing acoustic and electric inputs?

Functionally, neural responses are typically characterized either by the activity strength (i.e., the magnitude of the responses) that relates to the number and efficacy of the synapses (including the number of projections to the neurons) or by the structure of population activity, here, in particular, the synchronization of activity between neurons and structures (Buzsaki, 2006; Singer, 2021). The reason for the critical period in the congenitally deaf brain could be in effects on the response strength, on population synchrony, or both. Generally, the processing of the sensory input cannot be simply explained by the strength of the feedforward connectivity given that the fraction of thalamocortical connectivity represents <10% of all cortical synapses (Douglas and Martin, 2007; Winer and Lee, 2007), with ca. 20–30% of inhibitory synapses (Douglas and Martin, 2007; Winer and Lee, 2007; Markram et al., 2015). Therefore, it appears critical that sensory inputs are amplified by recurrent processing dependent on the priors stored in the circuits and thereby lead to widespread cortical activation and ultimately perception and behavior (Grossberg, 1987, 2013). Modeling studies confirm that recurrent interactions are key for preserving and propagating thalamic input (Binzegger et al., 2009). Congenital deafness interferes with the development of cortical synapses (Kral et al., 2005, 2009). It is thus likely that in congenital deafness the lack of sensory experience prevents sufficient recurrent processing and yields the cortex incapable of matching the sensory inputs to known sensory priors and features (Kral et al., 2017; Yusuf et al., 2021). This may account for the inability of the brain to assign meaningful interpretations to the sensory input.

Recurrent interactions both within and between populations of neurons comprising excitatory and inhibitory cells are generally known to lead to specific patterns of correlated and synchronized activity which can be separated in different frequency bands with specific functional correlates (Fries, 2005; Buzsaki, 2006; McGinley et al., 2015; Singer, 2021). It is also known that complementary information is conveyed by response magnitude and synchronized activity in other sensory systems like the visual cortex (McGinley et al., 2015; Peter et al., 2019; Singer, 2021). Sensory stimuli that cannot be predicted from the context may show an enhanced response magnitude since the synchronization is strongly influenced by the match of the sensory input and stimulus priors (Peter et al., 2019). Recurrent interactions are likely also critical for the integration between bottom-up sensory evidence with top-down predictions and are thought to play an important role in predictive processing (Bastos et al., 2012). Moreover, it is possible that response magnitude is key to sensory detection whereas the discrimination on input patterns depends on the precise structure of the high-dimensional neuronal activity vectors that result from recurrent cortical dynamics. The developmental formation of synapses allowing for functionally meaningful recurrent processing is known to be experience-dependent which naturally leads to their dysfunction in congenital deafness (Kral et al., 2005, 2019).

Here we investigated the differences in two aspects of neuronal activity, namely response magnitude and synchronization of activity. We furthermore compared congenital and acute deafness and acoustic and electric stimulation in anaesthetized cats. The electrical stimulation was conveyed by cochlear implants. The two electrically stimulated animal groups clinically translate to the postlingually deafened subjects at the moment of cochlear implantation (acute deafness group) and the prelingually deaf subject at the moment of late cochlear implantation (the congenitally deaf group).

We concentrated on the primary auditory cortex (field A1) and the secondary posterior auditory field (PAF). The fields are located along the “where” pathway of the feline auditory cortex (Lomber and Malhotra, 2008) and include feedforward (from A1 to PAF, Butler et al., 2017) and feedback projections (from PAF to A1, Barone et al., 2013) in both hearings and congenitally deaf cats. While a previous study concentrated on the analysis of functional connectivity between these fields using local field potentials (LFPs; Yusuf et al., 2021), reflecting the postsynaptic activity, the present study focused on a suprathreshold activity (unit responses) and due to its more local property additionally to the columnar interaction between layers of the primary auditory cortex.

## Materials and Methods

### Animals

**FIGURE 1:**
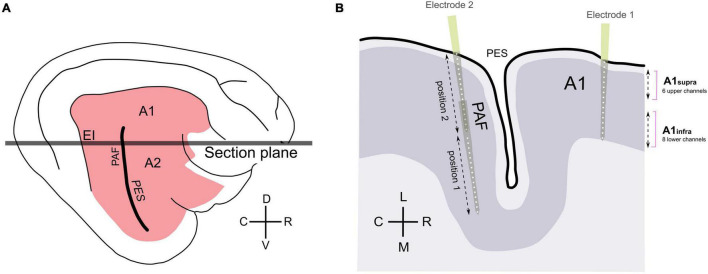
Schematics of recording positions. **(A)** Schematics of feline auditory cortex; the present experiments were performed in areas A1 and PAF. **(B)** Schematic illustration of the sectional plane of the auditory cortex from panel **(A)**, showing electrode penetrations in A1 and PAF. Based on histology, 6 upper channels in field A1 were grouped together (supragranular channels), and 8 lower channels in A1 were grouped together (infragranular channels). In PAF, penetrations down to 5,000 μm in two steps were used to densely map this field; each penetration included 32 recording sites in total. A1, primary auditory cortex; EI, intermediate area of the posterior ectosylvian gyrus; PAF, posterior auditory field; PES, posterior ectosylvian sulcus; D, dorsal; V, ventral; C, caudal; R, rostral; L, lateral; M, medial.

Five congenitally deaf cats (CDC) and eight hearing controls were used in the present study. We used animals reported on in a previous study (Yusuf et al., 2021); in contrast to the previous study, here we also analyzed the suprathreshold activity (action potentials). The experimental design included experiments on anaesthetized adult cats that were either congenitally deaf or had normal hearing. Stimulation was with sounds and electrical pulse trains presented through acutely implanted cochlear implants. Analyzed was the synchronization of spiking with LFPs activity within the primary auditory cortex and between a primary and a secondary auditory area (Figure 1A). The details of the experimental procedure were described in a previous publication (Yusuf et al., 2021) and will be briefly recapitulated here.

All cats were screened to select CDC from a colony of deaf white cats using acoustically-evoked brainstem evoked responses in the first month of life (Kral and Lomber, 2015). Additionally, the absence of hearing was confirmed at the beginning of the acute experiments. Eight normal controls were studied using acoustic stimulation (HA, four cats) and electric stimulation (HE, five cats). In one of the animals, we initially used acoustic stimulation and during the experiment switched to electric stimulation. For electric stimulation, intrascalar application of neomycin was used to destroy cochlear hair cells to prevent electrophonic responses (Sato et al., 2016). Therefore, the operational definition of hearing refers to the development of normal cochlear functions until the experiment, not to the cochlear functional state during the experiment.

The experiments were approved by the local state authorities and were performed in compliance with the Guidelines of the European Community for the care and use of laboratory animals (EUVD 86/609/EEC) and the German Animal Welfare Act (TierSchG).

### Experimental Procedures

All animals were premedicated with 0.25 mg atropine i.p. Initial anaesthesia was induced by 24.5 mg/kg ketamine hydrochloride and 2.1 mg/kg promazine phosphate. After tracheotomy, the animals were ventilated with 50% O_2_, 50% N_2_O, with the addition of 0.2–1.5% concentration of isoflurane (Lilly, Germany) to maintain anaesthesia (Kral et al., 1999). By keeping the burst-suppression index values within the range of 1–3 (Land et al., 2012), light anaesthesia levels were assured throughout the experiment. The temperature of the animals within 37.5–38°C was assured using a homeothermic blanket and a rectal temperature probe. The animal was monitored throughout the experiment using blood gas concentration, pH, bicarbonate concentration and base excess, glycaemia, oxygen saturation determined from the capillary blood. Ventilation was controlled using capnometry in the end-expiratory air. For further details, see also (Yusuf et al., 2021).

Subsequently, the animal’s head was fixed in a stereotactic frame (Horsley-Clarke). A small trephination at the vertex exposed the brain tissue, where a 1-mm silver-ball electrode was attached epidurally to record evoked auditory brainstem responses. The indifferent electrode used for the recordings was inserted medially into the neck muscles.

Hearing status was verified using auditory brainstem evoked responses (ABRs) with condensation clicks applied through a calibrated speaker (DT48, Bayer Dynamics, Germany) at levels up to 120 dB SPL (Otoconsult V2 low-impedance amplifier, 60 dB amplification; Otoconsult Filter F1, bandpass 0.01–10 kHz, amplification 40 dB, Otoconsult GmbH, Frankfurt am Main, Germany). Signals were digitized using a National Instruments MIO card (National Instruments, Munich, Germany), in which 200 sweeps were presented at a repetition rate of 33 Hz were averaged. For electrical stimulation, hair cells were destroyed by intrascalar application of 300 μl of neomycin sulfate that was washed out after 5 min. with Ringer’s solution. The total absence of ABRs confirmed the success of the procedure. The feline cochlear implant (MEDEL Comp. Innsbruck, Austria) had five contacts and has been described in detail previously (Yusuf et al., 2021). Stimulation was wide bipolar between the apical first and the fourth electrode (distance between active electrode: 3 mm). Electrically-stimulated controls and CDCs were implanted with a cochlear implant inserted *via* the round window. The implant was driven by optically-isolated current sources (CS1, Otoconsult, Frankfurt am Main, Germany). Electrically evoked auditory brainstem responses (E-ABR) to single biphasic pulses (charge balanced 200 μs/phase, repetition rate 33 Hz) were recorded and the lowest current levels evoke a brainstem response (E-ABR threshold currents) were determined.

Trephination was performed above the auditory cortex contralateral to the implanted ear and the dura was removed. The cortex was photographed. Using an ORIEL motorized x-y-z micromanipulator (1 μm precision in all directions), a silver-ball macroelectrode (diameter 1 mm) was positioned at a regular raster of nine cortical positions on the primary auditory cortex (field A1). The dorsal end of the posterior ectosylvian sulcus was used as a reference point. Signals (LFPs) recorded in response to a condensation click or an electric biphasic pulse applied through a cochlear implant (CI) were preamplified (60 dB, Otoconsult V2 low-impedance amplifier), amplified at a second stage (20 dB, Otoconsult Amplifier-Filter F1, filters 0.01–10 kHz), recorded using MIO cards and averaged (100 sweeps, repetition rate 1.97 Hz). The signals were stored and threshold current levels were evaluated at all recording positions with a precision of ±1 dB. These data were used to determine the cortical response threshold.

In and around the A1 region, the cortex was subsequently mapped using a microelectrode at a raster of 100–170 recording positions at the cortical surface (described in detail in Kral et al., 2009). This surface mapping identified the regions with the largest LFPs. Recordings were subsequently collected at these positions (“hot spots”; for details see Kral et al., 2009, 2013b). For this purpose a single-shank multi-electrode array (NeuroNexus, single shank, 16 contacts, spacing 150 μm, 177 μm^2^ contact area, electrode array length 2,400 μm, impedance 1–2MΩ) was used to penetrate A1 perpendicularly to the cortical surface to 2,400 μm depth, assuring recording from all cortical layers down to the white matter (Figure 1). A second array was used to map and register activity in field PAF. In PAF, the penetration was only possible parallel to the cortical surface. PAF penetrations were performed in two insertion steps: first, we penetrated to 5,000 μm depth, performed the recordings, and subsequently retracted the probe to 2,500 μm depth. At least one PAF penetration in each animal was marked by a fluorescent dye (DiI, 1,1-dioctadecyl-3,3,3′,3′-tetramethylindocarbocyanine perchlorate; Invitrogen) to allow histological reconstruction of the penetration track. For all recordings, the cortex was stabilized by a modified Davies chamber (Tillein et al., 2010). The reference electrode for recordings was a silverball electrode placed at the vertex epidurally.

### Stimulation and Recording

The stimuli analyzed in this manuscript were embedded in a pattern of other stimuli. The responses to the other stimuli were not analyzed in the present manuscript.

The contralateral ears were electrically stimulated by three biphasic electric charge-balanced pulses (200 μs/phase) presented through cochlear implants or acoustically stimulated by three condensation clicks (50 μs duration) presented through loudspeakers (repetition rate 500 pps, stimulus duration 4.4 ms). The stimulus presentation rate was 1/1,537 ms with 30 stimulus repetitions. Stimulation level was increased in 10 dB (acoustic) or 1–2 dB (electric) steps. Stimulation intensities were from at least 10 dB (acoustic) or 1 dB (electric) below the threshold to at least 60 dB (acoustic) or 9 dB (electric) above acoustic and electric ABR-threshold. In the present study, spike-field coherence was analyzed at 6 dB (electric) above the E-ABR threshold and 40 dB (acoustic) above the ABR threshold where the response strengths are in saturation.

For recording, signals were amplified by a 64-channel Cheetah amplifier (Neuralynx) with a gain of 5,000 and open filters (1–9,000 Hz), fed to a multifunctional data acquisition card (NI PCIe 6,259, National Instruments, Munich, Germany), 16-bit A/D converted at a sampling rate of 25 kHz per channel and stored on a computer.

### Histology

For each animal, at least one penetration for each field was marked by a fluorescent dye (DiI, 1,10-dioctadecyl-3,3,3′,3′-tetramethylindocarbocyanine perchlorate; Invitrogen). Since the probe attachment to the stereotactic frame was constant throughout the experiment, it was possible to extrapolate all penetrations directions from the stained and reconstructed tract. In PAF, histological reconstructions confirmed the correct location within this field in all animals reported.

After the experiments, the animals were transcardially perfused in deep anaesthesia. Following thoracotomy, 0.5 ml heparin (Heparin Natrium, Ratiopharm, Ulm, Germany) was injected into both ventricles. Two liters of 0.9% NaCl solution 2 L of fixative (4% paraformaldehyde) and 1 L of 10% sucrose were infused transcardially. The perfusion pressure was kept constant at 120–150 mmHg and monitored using the Perfusion One system (Leica Biosystems, Buffalo Grove, IL, United States). If required, the brain was postfixated in 4% paraformaldehyde and 10% sucrose overnight. For cryoprotection, each brain was placed in a 30% sucrose solution until it sank. Subsequently, the brain was blocked, frozen at −80°C, and cut at −20°C using a Leica Cryostat CM3050S (Leica Microsystems GmbH, Wetzlar, Germany) in section 50 μm thick. The sections were first photographed to reveal the DiI in fluorescent mode using a Keyence BZ-9000 microscope and subsequently stained using Nissl staining and SMI-32. For reconstruction, native fluorescence images were combined with the same Nissl-stained sections.

Layers in A1 were grouped into supragranular, granular, and infragranular based on the reconstructions of penetrations. The Nissl staining reveals the border of layer IV to layer V (Berger et al., 2017). Additionally, current source density measures (CSDs) that show a typical sequence of middle source in layer III and deep sink in layer V, with an initial sink followed by a source in layer IV between them (Kral et al., 2006), confirms this differentiation.

### Data Preprocessing

Analysis was performed on simultaneously recorded data from the two NeuroNexus probes located in auditory fields A1 and PAF. All contacts of the two probes are referred to as recording sites. We conducted offline computational analyses using MATLAB Mathworks 2021a (Mathworks Inc., Aachen, Germany) using the FieldTrip Toolbox (Oostenveld et al., 2011) and custom-made MATLAB scripts. Noisy recordings caused by unstable probe contacts, channels with artifacts, and occasional trials with spindles were not included in the analyses.

A linear interpolation was used to remove the artifacts of electric stimulation in post-stimulus recordings. Multiunit activity was subsequently extracted by band-pass filtering with Butterworth filter (2nd order edge frequencies 600–3,000 Hz with zero phase delay, using filtfilt function in MatLab). We removed the 50 and 100 Hz power line artifact from the signals using a discrete Fourier transform (DFT) filter. To further improve the signal-to-noise ratio in multiunit signals, the median of the probe at each trial was subtracted from each channel. We quantified unit activity following an automatic thresholding procedure by Quiroga et al. (2004) (as in Yusuf et al., 2017). Peristimulus time histogram (PSTH) with 2.5 ms unit responses binning were computed. A channel was considered significantly responding to the stimulus if the post-stimulus peak activity (0–50 ms) exceeded four times standard deviation (SD) from the mean pre-stimulus (baseline) activity (in what follows referred to as responsive sites). PSTHs of such responding channels were subsequently normalized relative to prestimulus and the relative firing rate (in dB) was then used to compute the grand mean PSTH.

To analyze the LFPs, the same recordings were down sampled: after using a low-pass filter to avoid aliasing (6th order Butterworth low pass filter with a frequency of 2,000 Hz), we down-sampled the signals to 500 Hz (corresponding to Nyquist frequency of 250 Hz). Additionally, the power line artifact was removed using 50 and 100 Hz DFT filters. The DC shifts in the baseline were subsequently removed in the time domain.

The resulting LFP recordings from primary auditory cortex layers were grouped based on the depth of the probe’s penetration corrected by the penetration angles in the histology and the histologically-determined layer limits (as in Yusuf et al., 2021). From two multielectrode arrays with 16 channels each, we categorized them into three groups: A1 supragranular layers (6 upper channels A1), infragranular layers (8 lower channels A1), and PAF (all 16 channels PAF) (see Figure 1B).

### Spike-Field Coherence

Prior to the spike-field coherence analysis, we performed a fast Fourier transformation using a multitaper method with a discrete prolate spheroidal sequence to analyze 8–32 Hz frequencies. This allowed a frequency-specific analysis of spike-field coherence. The analysis windows were set at 200–600 ms (400 ms duration). We deliberately avoided the time-locked evoked responses (representing the thalamic common input) by ignoring the first 200 ms post-stimulus.

To quantify the coupling between LFP and the spikes, we employed the pairwise phase consistency (PPC) method (Vinck et al., 2010, 2012). This method provides an unbiased measure (relative to the number of trials and recorded spikes, thus response “strength”). PPC was computed for the given LFP frequency using the formula


(1) $$P\,P\,C=\frac{2}{N.\left(N-2\right)}\,\sum_{j=1}^{N-1}\sum_{k=j+1}^{N}f\,\left(\theta_{j},\theta_{k}\right)$$

where *N* is the number of trials, *j,k* are the pairing iterators (i.e., the trials), and θ is the unity vector with the phase equal to the phase difference between the spike and the LFP at a given LFP frequency in the given trial pair. The function *f* computes the dot product of two vectors.

Computation objects in PPC are paired vectors, where PPC averages the dot product for all available pairs. Results yielded by PPC reflect the true angle distribution, where a higher PPC denotes smaller angular distance, where 1 is maximum coherence and 0 no coherence. Furthermore, the “surrogate” spike-field coherence was computed by shuffling the trials. Subtracting the shuffled PPC from the non-shuffled thereby removes any remaining time-locked stimulus component and thus eliminates the common input from the result.

### Grand Mean PPC

Rayleigh statistics were computed to measure uniformity in circular data of the PPC. Multiple comparisons were accounted for by the false detection rate procedure (Benjamini and Yekutieli, 2001). If the phase distribution was non-significant, we discarded the channel pairs.

From the remaining pairs, the grand mean PPC was computed, grouped into Hearing Acoustic (HA), Hearing Electric (HE), and Deaf Electric (DE). In all groups, we measured the average value of PPC from and to each anatomical region (A1 supra, A1 infra, and PAF). Pairwise comparison of PPC spectra was computed using the two-tailed Wilcoxon rank-sum test (false discovery rate corrected, *p* < 0.001). The statistical significance of the mean difference between HA-HE and HE-DE at 10 Hz was compared using a two-tailed *t*-test.

### Phase Distribution of Intrinsic Coupling

Avoiding the spillover from the other frequency band, we analyzed the center frequency of the alpha band (*f* = 10 Hz). The 10 Hz alpha PPC data was further analyzed to assess the phase distribution. We constructed the probability histogram on repeated periods of phase angles. To see the phase distribution difference between A1 layers in the three groups, we compared A1 supra-to-supra and A1 supra-to-infra spike-field PPC angular phase by finding the angular mean. To justify our findings, we permuted the indices for all data. We computed *p*-value as the number of events where the mean difference in permuted data was higher than non-permuted data, divided by the number of permutations (*n* = 1,000), multiplied by the number of groups (*k* = 3). Multiple comparisons were accounted for by Bonferroni correction.

## Results

We recorded three groups of animals: hearing acoustically stimulated cats (HA), hearing electrically stimulated cats (HE), and congenitally deaf cats (stimulated electrically, CDC). The electrically stimulated animals had no prior experience with the electric stimulus, it was used acutely only to test the function of the auditory cortex. The acoustic stimulation was a train of three condensation clicks (50 μs duration) and the electric stimulation was a train of three biphasic charge-balanced pulses (200 μs/phase). The repetition rate was always 500 pps, yielding a stimulus overall duration <5 ms. To avoid electrophonic responses (Sato et al., 2016) hair cells in hearing electrically stimulated animals were removed by intracochlear application of Neomycin before cochlear implantation. The congenitally deaf animals do not have any surviving hair cells (Heid et al., 1998). The three groups allow two types of comparisons: the HA–HE comparison yields information on the effect of stimulus mode (acoustic vs. electric, familiar vs. unfamiliar). The comparison between HE and CDC yields information on the effect of developmental hearing experience on the responses.

Recordings in A1 were made perpendicularly to the cortical surface, providing layer-specific data. The recordings were separated into those from supragranular and infragranular layers, as in previous studies (Yusuf et al., 2021, see section “Materials and Methods”). Additionally, recordings were performed in the PAF, a secondary auditory field directly connected to A1 in both hearing and congenitally deaf cats (Barone et al., 2013; Butler et al., 2017). Due to PAFs anatomical location on the caudal bank of the posterior ectosylvian sulcus, recordings were tangential to the layers and therefore layer differentiation was not possible (Figure 1).

**FIGURE 2:**
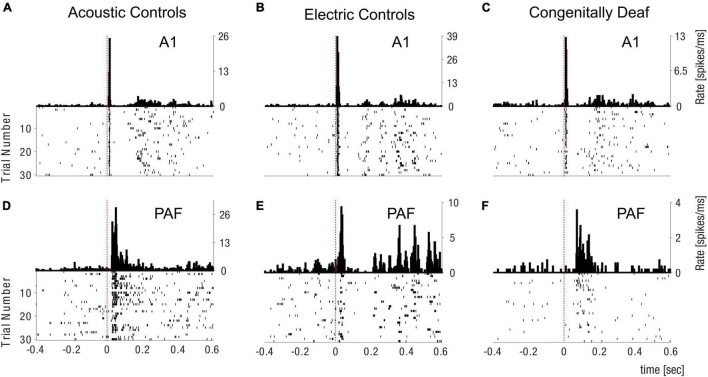
Peristimulus histogram and spike raster plot examples. Examples of peristimulus histograms (PSTHs) (above) and spike raster plot (below) in the field A1 **(A–C)** and PAF **(D–F)** for Acoustic Controls **(A,D)**, Electric Controls **(B,E)**, and Congenitally Deaf Cats **(C,F)**. PSTHs are shown in spikes/millisecond, dashed red lines are showing the stimulation time point. A1 primary auditory cortex; PAF posterior auditory field.

**FIGURE 3:**
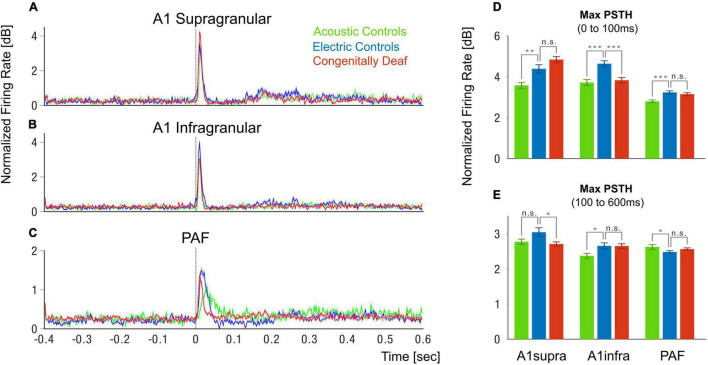
Peristimulus histogram grand mean. **(A–C)** Grand mean of the peristimulus histograms (PSTHs) in supragranular layers of A1 **(A)**, infragranular layers of A1 **(B)**, and posterior auditory field **(C)**. PSTHs are plotted in normalized firing rate (dB relative to mean- prestimulus). Shaded areas show the standard error of the mean. **(D–E)** Bar plots of PSTH maximum for the three groups and three different areas and layers for the early time window (0–100 ms), **(D)** and late time window (100–600 ms), **(E)**. Green represents Acoustic Controls, blue represents Electric Controls, and red represents Congenitally Deaf Cats. N.s. not significant, *p < 0.05, ^**^p < 0.01, ^***^p < 0.001, *t*-test statistics.

We first analyzed the firing rate responses to the different types of stimulation and the different layers in A1. Multiunit activity was computed by bandpass-filtering the signals, denoising them by removing the median across channels, and subsequently finding spikes by thresholding the signals (see section “Materials and Methods”). Examples of raster plots of the data are shown in Figure 2. We subsequently computed peristimulus time histograms, normalized them to the prestimulus period, and analyzed the magnitude of such normalised firing rate responses in both the early onset and late response time window (Figure 3A; Yusuf et al., 2017). Altogether, for the peristimulus time histograms, we analyzed 470 recording sites (i.e., electrode contacts) in HA, 586 in HE, and 798 in CDC. Nonresponsive units were defined as showing no significant change in firing properties after the stimulus. Nonresponsive unit proportion in HA was 37% in supragranular layers, 24% in infragranular layers, and 73% in PAF. With the stronger, hypersynchronized electric stimulation (HE) there were 18% non-responsive units in supragranular layers, 10% in infragranular layers, and 61% in PAF. In comparison to HE, CDCs had fewer nonresponsive units in A1 supragranular layers (6%), but had more nonresponsive units in A1 infragranular layers (24%) and PAF (73%). All subsequent analyses were performed on the responsive sites.

The PSTH response in the HA was in some measures weaker than in HE, likely due to the strong synchrony of the electrically-evoked activity in the auditory nerve (Kral et al., 2021). We observed no significant difference of the PSTH maximum response between the HE and CDC groups in the early onset response (0–100 ms) in the A1 supragranular layers and PAF (Figure 3D), nor in the late-onset response (100–600 ms) in the A1 infragranular layers and PAF (Figure 3E). Significant reductions of responses in CDC compared to HE were only found in the early onset A1 infragranular layers (Figure 3D) and late-onset A1 supragranular layers (Figure 3E). These analyses thus show that the CDC group does preserve responsiveness to the electric stimulation. The responsiveness in many measures (supra A1 early, infra A1 late, early PAF, and late PAF) was not different from HE animals.

Therefore, we wondered whether the three groups differ in the synchronized neuronal activity, which is a hallmark of recurrent processing in the cerebral cortex. To investigate this, we used a previously established approach to compute spike-field coherence indicating the extent to which spikes are synchronized to the post-synaptic activity in the local area (Buzsáki and Schomburg, 2015; Pesaran et al., 2018). This approach has, compared to LFP analyses, the advantage that it strictly quantifies synchronization in local circuits (e.g., avoiding volume conduction) while at the same time providing a more sensitive measure than spike-spike correlations. To quantify spike-field coherence we used the PPC measure that is unbiased for the number of spike discharges. The number of site pairs analyzed was several hundred for all possible pairs (see Table 1).

**TABLE 1:** Total site-pairs with significant spike-field coherence coupling.

	Acoustic control			Electric control			Congenital deaf		
	A1 Supra	A1 Infra	PAF	A1 Supra	A1 Infra	PAF	A1 Supra	A1 Infra	PAF
**A1 Supra**	648 (51%)	864 (69%)	1,728 (28%)	972 (70%)	1,296 (76%)	2,592 (23%)	1,512 (72%)	2,016 (61%)	4,032 (12%)
**A1 Infra**	864 (47%)	1,152 (70%)	2,304 (27%)	1,296 (66%)	1,728 (76%)	3,456 (23%)	2,016 (23%)	2,688 (70%)	5,376 (11%)
**PAF**	1,728 (40%)	2,304 (57%)	4,608 (46%)	2,592 (57%)	3,456 (64%)	6,912 (31%)	4,032 (56%)	5,376 (42%)	10,752 (24%)

*Numbers are shown in N total site-pairs followed by percentage (in brackets) of significant spike field coherence coupling.*

**FIGURE 4:**
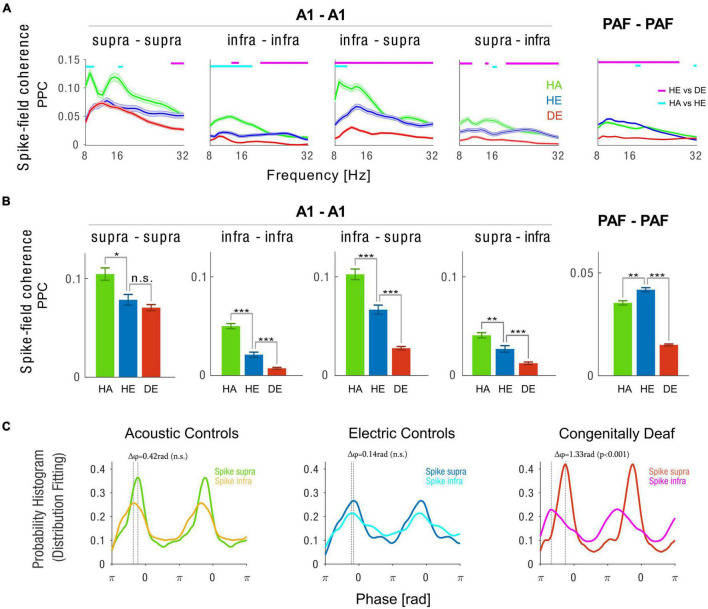
Intra-areal spike-field coherence in A1 and PAF. **(A)** Spike-field coherence spectra computed with pairwise phase consistency (PPC) method for A1 supragranular spikes–A1 supragranular LFPs (first column), A1 infragranular spikes–A1 infragranular LFPs (second column), A1 infragranular spikes–A1 supragranular LFPs (third column), A1 supragranular spikes–A1 infragranular LFPs (fourth column), and PAF spikes–PAF LFPs (fifth column). Shaded areas show the standard error of the mean. Statistical pairwise comparisons are shown for electric control vs. deaf (magenta line above the graph) and animals with intact cochleae vs. acutely deafened cochleae (cyan line above the graph) using the two-tailed Wilcoxon rank-sum test (false discovery rate corrected, *p* < 0.001). **(B)** Bar plots of 10 Hz alpha spike-field coherence are plotted in the same order as in panel **(A)**. HA (green) Acoustic Controls, HE (blue) Electric Controls, DE (red) Congenitally Deaf Cats. n.s. not significant, *p < 0.05, **p < 0.01, ***p < 0.001, *t*-test statistics. **(C)** Probability histogram using fitting distribution showing phase preference from PPC phase distribution for the three animal groups, each for A1 supragranular spikes–A1 supragranular LFPs and A1 infragranular spikes–A1 supragranular LFPs. The difference between two probability histograms was tested using a permutation test (*n* = 1,000). A1 primary auditory cortex, PAF posterior auditory field, LFP local field potentials.

We first determined the significantly coupled sites (Rayleigh statistics, see section “Materials and Methods”); only these were used for subsequent analysis. In general, more than half of all electrode pairs showed significantly coupled sites within A1. This proportion was smaller within PAF and between A1 and PAF (Table 1). For analysis between 333 and 2,530 significantly coupled recording site pairs remained, most recording site pairs were found in PAF-PAF in the CDC group and the least recordings site pairs were in A1 supra-supra in the HA group. The spike-field coherence was subsequently analyzed in the late response (200–600 ms time window) in order to avoid correlations that may have arisen because of the time-locked evoked responses (Figures 4A,B). Nonetheless, we subtracted the “surrogate” spike field coherence which was computed by shuffling the trials, thereby removing any time-locked stimulus component. Spike field coherence was determined between all coupled sites; different laminar components were separated. The spike-field coherence in the prestimulus time did not provide qualitatively different outcomes compared to the late poststimulus PPC (shown in Supplementary Figure 1).

While responses were generally weaker in the acoustic condition (Figure 3), we found a stronger synchronization in acoustic stimulation compared to electric stimulation in controls (Figure 4, green vs. blue lines and bars). A1 to A1 synchronization was significantly stronger in the HE than in CDCs (Figure 4, blue vs. red lines and bars). This effect was particularly prominent for the coupling between the spiking of A1 infragranular neurons with A1 supragranular LFPs (Figures 4A,B, blue vs. red lines and bars). This matches the findings that there are anatomical deficits in CDCs particularly prominent in infragranular layers (Berger et al., 2017). Interestingly, the connection between supragranular and infragranular layers in A1 was asymmetric. Compared to the A1 infra-supra connection (Figure 4B third column), the supra-infra connection (Figure 4B fourth column) was weaker in all studied animal groups (supra infra vs. infra-supra (mean ± SEM) for HA group 0.046 ± 0.003 vs. 0.105 ± 0.005, *p* < 0.001; HE group 0.026 ± 0.003 vs. 0.048 ± 0.004, *p* < 0.001; CDC group 0.012 ± 0.001 vs. 0.021 ± 0.002, *p* < 0.001, two-tailed *t*-test statistics). We furthermore observed that synchronization within PAF was significantly reduced in the CDCs group as compared to the HA and HE groups.

To further investigate the interaction between A1 supragranular and infragranular layers we examined the phase distributions of spiking discharges relative to the LFPs (Figure 4C). In both HA and HE groups, we found no significant difference in the preferred phase of synchronization between infragranular and supragranular layers (Δ = 0.42rad with *p* = 0.072 for HA and Δ = 0.14rad with *p* = 0.318 for HE, permutation test with *n* = 1,000). Strikingly, however, we found that in the CDC group there was a large and significant phase difference in the preferred phase of spiking discharges between A1 supragranular and infragranular layers (Δ = 1.33rad with *p* < 0.001, permutation test with *n* = 1,000). Together these findings document that congenitally deaf cats have a strong deficit in both local A1 and PAF synchronization and there is a decoupling between the infra and supragranular layers.

**FIGURE 5:**
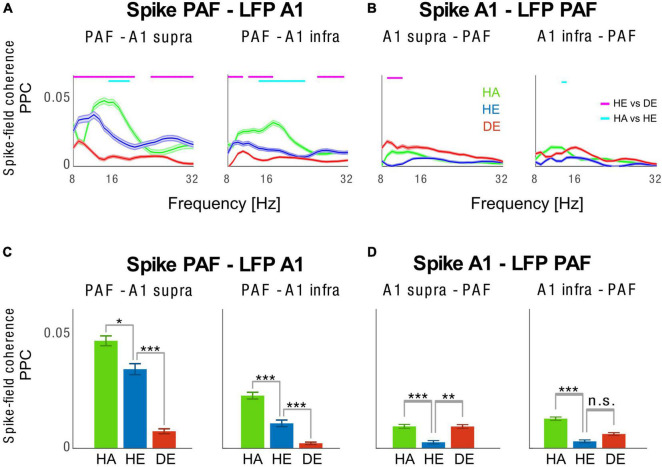
Inter-areal spike-field coherence between A1 and PAF. **(A)** Spike-field coherence spectra computed with pairwise phase consistency (PPC) for PAF spikes–A1 supragranular LFPs (left panel) and PAF spikes–A1 infragranular LFPs (right panel). Shaded areas show the standard error of the mean. **(B)** Spike-field coherence spectra were computed with PPC for A1 supragranular spikes – PAF LFPs (left panel) and A1 infragranular spikes–PAF LFPs (right panel). Statistical pairwise comparisons are shown for electric control vs. deaf (magenta line above the graph) and animals with intact cochleae vs. acutely deafened cochleae (cyan line above the graph) using the two-tailed Wilcoxon rank-sum test (false discovery rate corrected, *p* < 0.001). **(C,D)** Bar plots of spike-field coherence are plotted in the same order as in panels **(A,B)**, representing the frequency of 10 Hz alpha. HA (green) Acoustic Controls, HE (blue) Electric Controls, DE (red) Congenitally Deaf Cats. n.s. not significant, *p < 0.05, **p < 0.01, ***p < 0.001, two-tailed *t*-test statistics. A1, primary auditory cortex; PAF, posterior auditory field; LFP, local field potentials.

Finally, we analyzed synchronization between spikes and LFPs among PAF and A1 pairs (Figure 5). We have previously shown that LFP-LFP connectivity between A1 and PAF is reduced in CDCs as compared to HE and HA groups and that the PAF to A1 communication in the late response predominantly occurs in feedback direction (Yusuf et al., 2021). Here we asked how the synchronization between spikes and LFPs differed between the three groups, which provides enhanced spatial localization compared to LFP-LFP analyses. We found that there was an asymmetry in the coupling in spike and LFPs between A1 and PAF in HA condition. In particular, we found that PAF spikes were strongly synchronized to A1 LFPs, but that coupling was significantly weaker between A1 spikes and PAF LFPs. A similar pattern was observed for the HE condition. By contrast, coupling between PAF spikes and A1 LFPs was strongly reduced in the CDC group, with the lack of asymmetry in the interareal coupling. These findings show an operation of recurrent interaction between PAF and A1 in hearing animals in both acoustic and electric stimulation, but a loss of A1–PAF synchronization in CDCs.

## Discussion

**FIGURE 6:**
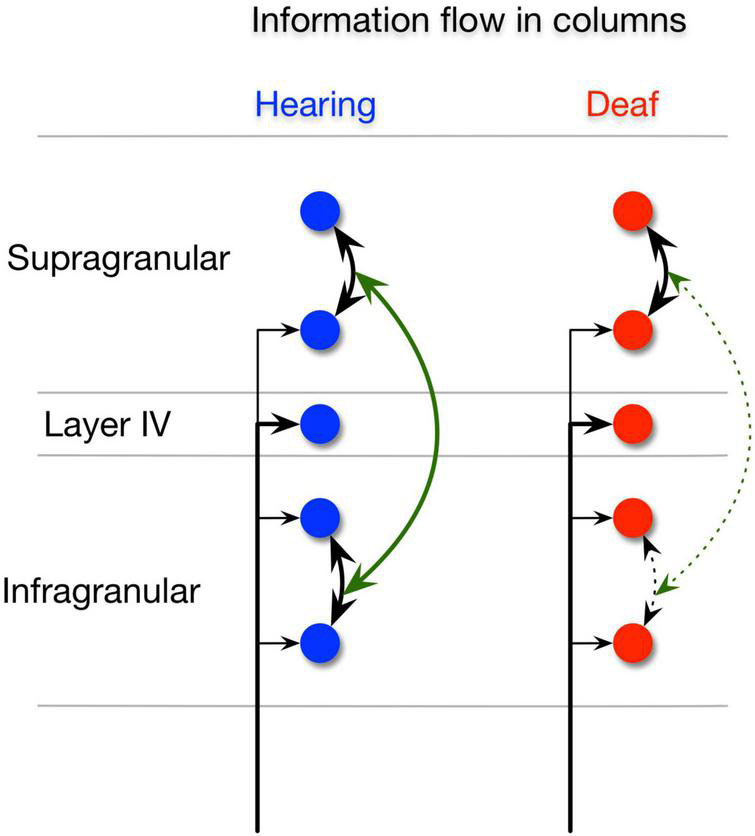
Intrinsic information flow in the hearing and deaf animals. Illustration of intrinsic information flow between supragranular and infragranular layers in the hearing animals (left), (blue) showing normal strong supra-supra, infra-infra, and supra-infra interactions. Congenitally deaf animals (right), (red) show normal supra-supra interactions but significant deficits in the supra-infra and infra-infra interactions.

In this study, we compared auditory responses between congenitally deaf, acutely deafened, and hearing cats. We asked which aspects of neural activity distinguish these three groups of animals. In this particular experiment, we found no systematic tendency of evoked unit responses to be weaker in CDC and stronger responses in electric compared to acoustic stimulation. However, we found that in CDC there was a prominent reduction both within-areal A1 and PAF synchronization and in inter-areal synchronization, and that there was a decoupling between supra and infragranular layers in CDCs (Figure 6). Because synchronized activity results from recurrent interactions among populations of excitatory and inhibitory neurons, these findings suggest that deficits in recurrent processing are characteristic deficits of congenital deafness.

Recurrent processing is a hallmark of the cerebral cortex and likely essential to all forms of cortical computations and, ultimately, perception (for review, see Singer, 2021; Vezoli et al., 2021). It is widely believed that sensory processing results from an integration of the bottom-up sensory inputs and the stimulus priors that are stored in the recurrent connections within and between cortical areas, as well as sensory predictions that the cortex can make based on the spatiotemporal context. In general, there are two views on the way in which bottom-up sensory evidence interacts with sensory priors. On one hand, the key computational role of the cortex is to discriminate fine differences in sensory patterns and transform these into perceptual phenomena. It is thought that recurrent processing is key to the discrimination and extraction of sensory input patterns a process that can be thought of as an attractor dynamic. Even relatively weak input patterns that likely comprise a small fraction of synaptic inputs to the cortex can lead to the widespread propagation of activity across cortical areas and ultimately behavioral responses and perceptual discriminations. This likely depends on the amplification of weak bottom-up inputs by the high-dimensional recurrent interactions between neuronal populations (Raizada and Grossberg, 2003; Grossberg, 2013; Singer, 2021). The recognition and amplification of sensory input patterns likely depend on experience-dependent synaptic weight distributions. Another view on sensory processing is the predictive coding framework (Friston, 2010; Keller and Mrsic-Flogel, 2018) which rather postulates that those patterns of activity that do not match sensory priors are amplified and are not explained away by top-down feedback. In this view surprising bottom-up inputs that do not match priors and contextual predictions should lead to enhanced sensory responses.

We can think of our three groups of animals as follows: the HA group we provide with the naturalistic sensory stimulation which likely matches both innate and stimulus-dependent priors. One can expect that in these animals the recurrent cortical connections are tuned to the naturalistic auditory experience. In the HE group, we expect that the animals have learned the sensory priors about the natural acoustic inputs and are able to recognize and discriminate different acoustic input patterns. The electrical cochlear stimulation can now be conceived as an off-manifold sensory perturbation. One would expect that this novel sensory input pattern can initially not be matched to the existing sensory priors while at the same time providing a salient prediction-error-like input pattern. For the CDC we would expect that there is an absence of sensory priors about the natural sensory stimuli and that the cortex is not able to match the electrical stimulation to any known input pattern. The present results are consistent with such expectations and consistently demonstrate that our measure is sensitive enough to reveal differences in stimulus mode and developmental experience.

A particular advantage of the present study is the animal model used since a lot of information on cochlear implant responses has been presented for CDCs. The two studied auditory fields are distant but directly connected by fiber tracts in both hearing and congenitally deaf cats (Barone et al., 2013; Butler et al., 2017). The columnar organization in the cat allows direct transfer to the primate cortex, which is different for rodents (Espinosa and Stryker, 2012). Finally, the cat highly depends on hearing in its natural condition and thus has a highly developed auditory system with 13 auditory areas (Winer and Lee, 2007). The functional properties of the 13 areas, including the secondary field PAF, have been described in detail before (Phillips and Orman, 1984; Kitzes and Hollrigel, 1996; Heil and Irvine, 1998; Loftus and Sutter, 2001; Harrington et al., 2008). Last but not least cortical responses to the cochlear implant have been studied in detail both in hearing and in congenitally deaf cats, both in A1 (Hartmann et al., 1997; Raggio and Schreiner, 1999; Kral et al., 2006, 2009, 2013b) and in PAF (Yusuf et al., 2017, 2021).

One methodological aspect is the orientation of penetrations: they were perpendicular to the cortical surface in A1 but tangential in PAF. This is given by the anatomy of these fields, where PAF is partly hidden in the caudal bank of the posterior ectosylvian sulcus. This not only precluded layer-specific analyses in PAF, but it also potentially affected the PSTH comparisons between A1 and PAF shown in Figure 3. Furthermore, the field PAF was actually mapped with many penetrations of the NeuroNexus probe along its dorsoventral axis, whereas in A1 the probe was placed only in the hot spots. However, the procedures were exactly the same in all three groups of animals, and therefore this did not affect the connectivity comparisons between the three studied animal groups.

The stimuli used were presented repeatedly in these experiments; however, stimulation was embedded within a large set of diverse stimuli (not analyzed in the present study). To additionally prevent any habituation or stimulus-specific adaptation phenomena (Pérez-González and Malmierca, 2012), stimuli were presented at a very slow presentation rate (0.59 Hz). Therefore, we did not find systematic response adaptation phenomena in this study.

The spike-field coherence that we observed in the late response window was, in the general pattern of group differences, also replicated in prestimulus time connectivity (Supplementary Figure 1). This supports the concept that spontaneous activity reflects aspects of stimulus-related activity (Arieli et al., 1996; Berkes et al., 2011). It is, however, important also to point to the differences between the stimulus-related and ongoing activity, and the respective functional connectivity (see e.g., Yusuf et al., 2021): (i) we did not show the early poststimulus time window where differences to spontaneous activity and connectivity were larger and (ii) in the present analysis we subtracted the evoked part from the coherence. This means that we essentially analyzed induced-activity-related connectivity. Last but not least, there were differences in the detailed patterns of spike-field coherence between the prestimulus and the late poststimulus activity (Supplementary Figure 1) as visualized also in stimulus-related field-field coherence (Yusuf et al., 2021).

The present results overall fit well into previous data on congenitally deaf cats (Kral et al., 2006; Tillein et al., 2010). With respect to the general responsiveness the present study showed similar responsiveness in the CDCs as in HE in supragranular layers, but weaker responsiveness in infragranular layers, as in a different set of animals in Kral et al. (2006). The present study excluded layer IV from the analysis that yields the strongest late response in unit activity in hearing controls (Hajduk et al., 2018). As previously demonstrated on a different set of animals (Tillein et al., 2010), the CDCs showed less responsive units than HE.

With respect to spike-field coherence, the present data revealed stronger intrinsic (within area) connectivity compared to extrinsic connectivity, as would be expected for a small-world network with predominant within-column local connectivity (Strogatz, 2001; Hilgetag and Kaiser, 2004; Harris and Shepherd, 2015). The general rule is that with increasing distance connection strength decreases (Cossell et al., 2015). The present results of A1–PAF connectivity in relation to intrinsic A1 connectivity reflect this rule well. Finally, the dependence of connectivity from developmental experience might increase with increasing distance between recording sites. Therefore, it is possible the present results in CDCs are co-affected by such dependence.

The functional connections were often asymmetric: while the spikes in infragranular layers did synchronize more the LFPs in supragranular layers in controls, the effect was much weaker in the reverse direction. Also, while spikes in PAF did synchronize the LFPs in A1 in hearing controls, the general coupling was much weaker in the reverse direction. While spikes in PAF synchronized the LFPs in A1 less in CDCs, in the reverse direction this was not the case (there the synchrony was weak, Figure 5, and the angle distribution was random, data not shown). The data on interareal spike-field coherence was in general agreement with the previous detailed analysis of the LFP-LFP synchronization between A1 and PAF in hearing and deaf cats performed on the same animals (Yusuf et al., 2021). The replication of the previous outcome supports the concept that the measures used in these studies do well reflect functional connectivity. Additionally, spike-field coherence with the more local signals allowed us to analyze within-areal connectivity which was not the focus of the previous study. Regarding that, we could observe that in deaf cats the interaction of the supra-to-infragranular layers, essential for the interaction between bottom-up and top-down streams of information, is decoupled.

Cochlear implants are the most successful neuroprosthetic device (Kral et al., 2021). While CIs are exceptionally successful in restoring speech understanding in adult, postlingually deaf subjects, CI leads to poor speech comprehension and deficits in feature sensitivity in congenitally deaf adults (Busby et al., 1993; Busby and Clark, 1999; Rousset et al., 2016; Rousset, 2017). In contrast, CI in congenitally deaf infants has a high success rate when implantations are performed within the first 3 years of life (Manrique et al., 1999; Niparko, 2010; Karltorp et al., 2020). Similar critical periods were observed with CI stimulation in CDCs (Kral et al., 2006, 2013a,b) and related to pronounced synaptic pruning in the cortex of CDCs (Kral et al., 2005; Kral and Sharma, 2012).

Our hypothesis is that the low success rate of CI in congenitally deaf is also because the novel sensory input patterns cannot be matched to any priors as the recurrent cortical connections are not tuned to discriminate and extract different sensory input patterns. On the other hand, when humans become deaf at a late age the cortical circuits are likely capable of extrapolating a known sensory prior to the novel electric input patterns by fine-tuning the existing synaptic weight distributions. In this study, we found evidence for deficits in recurrent processing in CDCs which was reflected by the lack of synchronized activity within and between cortical areas. However, the electric stimulation generally provided a relatively strong sensory response both in congenitally deaf and acutely deafened animals, which suggests that it is not per se the amplitude of the sensory responses but rather the recurrent processing of sensory input that distinguished the different groups. This is in principle compatible with predictive coding in that sensory inputs that cannot be matched with priors evoke strong responses. Predictive coding further postulates that the connection of supragranular to infragranular layers is key to the integration of bottom-up to top-down signals given the known anatomical layer segregation between bottom-up to top-down projections (Rouiller et al., 1991; Markov et al., 2014; Vezoli et al., 2021). Thus, the observed decoupling between supragranular and infragranular layers likely entails a deficit in the integration of bottom-up and top-down signals. These findings are in agreement with our previous work where we examined the A1-PAF LFP-LFP connectivity with PPC and Granger causality (Yusuf et al., 2021). In that study, we observed that there was a lack of interareal coherence between A1 and PAF during the stimulus period as well as a reduction in synchronization of alpha and beta activity in the late phase of the stimulus period. Also in the data in the current study, we found evidence for a reduction in top-down feedback from PAF to A1 in CDCs. Feedback projections develop later than feedforward projections in the visual system (Barone et al., 1996; Katz and Shatz, 1996; Batardière et al., 2002). This could explain the difference in their susceptibility to developmental sensory experience.

If this should hold for the auditory system, too, then it would be plausible why cortical top-down connectivity is affected more than bottom-up connectivity. However, the present study cannot disentangle whether it is the absence of priors that reduces the influence of PAF on A1, whether the neuronal connection between PAF and A1 is generally weakened, or whether both these effects participate. Given that postnatal synaptic development in A1 is massively affected by congenital deafness (Kral et al., 2005; Kral and Sharma, 2012), and given that the present experiments were performed in anaesthetized animals where the role of attention is absent, we assume that it is the underlying connection of PAF to A1 that is weakened. Nonetheless, anaesthesia reduces the interaction between supragranular and infragranular layers (Suzuki and Larkum, 2020). Therefore here the observed differences between the animal groups in anaesthesia are very likely underestimating the true effect sizes.

Another way to interpret these specific functional differences between congenital and acute deafness is that the cortex in CDC is still capable of simple detection, which likely relies on response magnitude, but is unable to discriminate fine patterns, which depend on recurrent interactions in high-dimensional neuronal space (Kral et al., 2019). Consistent with this interpretation, the late-implanted congenitally deaf patients are able to detect the stimulation, but are not able to discriminate and assign meaningful perceptual interpretations to the stimulation patterns. Consistent with this interpretation is also the loss of feature sensitivity in CDCs reported in previous studies (Tillein et al., 2010, 2016).

The described functional deficits likely have an anatomical correlate that may be particularly prominent in the infragranular layers. We have previously shown that there is a shrinkage of the infragranular compartment of CDCs which was not observed in supragranular layers (Berger et al., 2017). This may potentially reflect a reduction in the dendritic arborization of myelination patterns in the infragranular layers.

In previous studies, we have shown that if CDCs learn to associate a stimulus with a reward there is an amplification of responses with experience (Kral et al., 2006, 2013b,a). Previous studies using current source density analysis documented in CDCs a reduction of activity in the cortical column of A1 particularly pronounced in deep layers. In this study with unit responses, there were a smaller fraction of responsive units in CDCs (similar to Tillein et al., 2010) and we found that the evoked responses in the infragranular layers were slightly reduced. However, supragranular layers were partially spared of this effect. A possible hypothesis is that activity in the infragranular layers has a modulating suppressive effect on supragranular layers (Olsen and Winder, 2012) and that the decoupling of infra and supragranular layers account for the partial increase in the supragranular activity in CDCs. Cortical microstimulation in deep layers of the auditory cortex alone also did not activate supragranular layers in hearing guinea pigs but increased cortical induced responses to an acoustic stimulus (Voigt et al., 2017, 2018).

Future work should address in detail how signals propagate across a large number of brain regions and in addition investigate the activity patterns of distinct cell types. It is known that subclasses of GABAergic neurons are affected by developmental hearing loss (Kotak et al., 2005, 2008; Mowery et al., 2015). In particular, VIP and SOM neurons play an important role in recurrent processing and the integration of sensory inputs with behavioral context (Batista-Brito et al., 2017). Furthermore, the experience-dependent formation of synapses is likely gated by the activity of specific subclasses of GABAergic neurons, in particular SOM and VIP positive neurons (Harris and Shepherd, 2015; Blackwell and Geffen, 2017). A previous study has shown that deficits in VIP interneurons lead to a similar phenotype as we observed in CDCs, in particular a loss of synchronization among excitatory cells which were accompanied by deficits in sensory learning (Batista-Brito et al., 2017). Thus, it is possible that the activity of specific classes of neurons is down- or up-regulated in CDCs. This may provide an interesting target for clinical interventions in the future. Another hypothesis we derive from this work is that acoustic stimulation in hearing subjects should give rise to much more widespread cortical activity (over more cortical areas) when compared to electric stimulation. That can be tested by performing electrophysiological recording across a large number of areas (comp. Yusuf et al., 2017). Previously we have shown a slow but extensive increase in responsiveness in field A1 of CDCs chronically stimulated with a CI (Klinke et al., 1999), particularly with early cochlear implantation (Kral et al., 2006, 2013a,b; Kral and Sharma, 2012). We expect that the same holds true for higher-order auditory areas and that following an early CI there would be a corresponding reorganization with experience.

## Data Availability Statement

The original contributions presented in the study are included in the article/Supplementary Material, further inquiries can be directed to the corresponding authors.

## Ethics Statement

The animal study was reviewed and approved by “Niedersächsisches Landesamt für Verbraucherschutz und Lebensmittelsicherheit” of the Government of the State of Lower Saxony, Oldenburg, Germany.

## Author Contributions

AK designed the project and experiments and obtained the funding. PH, JT, and AK performed the experiments. PY and AL analyzed the data with the supervision of PH, AK, and MV. PY, AL, and AK prepared the figures. MV and AK drafted the first version of the manuscript. PY, AL, MV, PH, and AK edited the manuscript. All authors approved the manuscript.

## Conflict of Interest

JT was employed by the company MEDEL. The remaining authors declare that the research was conducted in the absence of any commercial or financial relationships that could be construed as a potential conflict of interest.

## Publisher’s Note

All claims expressed in this article are solely those of the authors and do not necessarily represent those of their affiliated organizations, or those of the publisher, the editors and the reviewers. Any product that may be evaluated in this article, or claim that may be made by its manufacturer, is not guaranteed or endorsed by the publisher.
