# Supplementary material for: Deficient Recurrent Cortical Processing in Congenital Deafness
Source: Front Syst Neurosci. 2022 Feb 25;16:806142. doi: 10.3389/fnsys.2022.806142 (PMC8913535; doi:10.3389/fnsys.2022.806142)
Supplement: Supplementary file 1 [file Data_Sheet_1.PDF]

SUPPORTING MATERIALS

Supplementary Figure 1

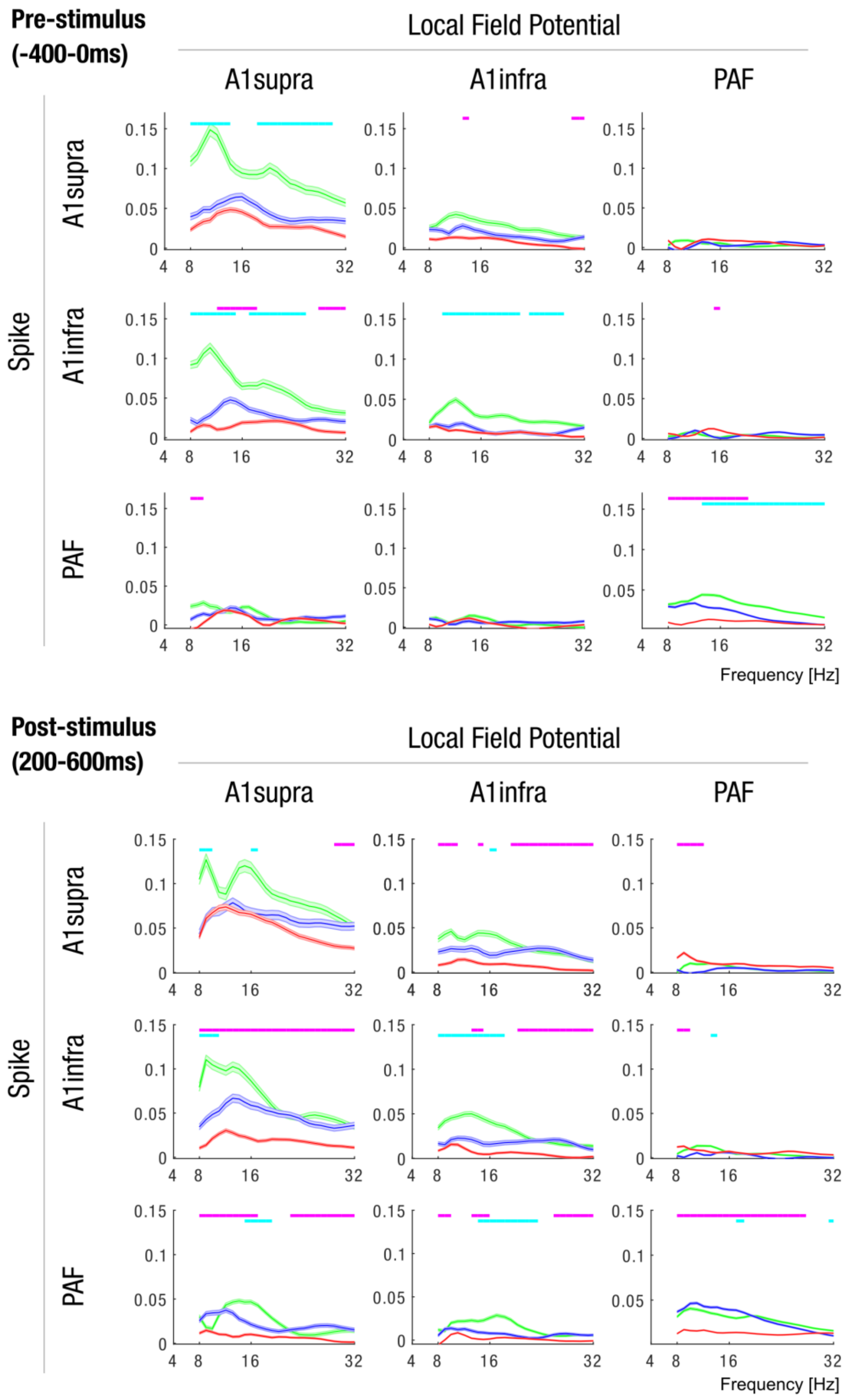

Supplementary Figure 1. Spike-field coherence matrices for prestimulus time (-400 to 0 ms, top) and poststimulus time (200 to 600 ms, bottom).

Spike-field coherence spectra computed with pairwise phase consistency (PPC) method for showing the intra-areal in A1 and PAF, and inter-areal between A1 and PAF. HA (green) Acoustic Controls, HE (blue) Electric Controls, DE (red) Congenitally Deaf Cats. Statistical pairwise comparisons are shown for electric control vs. deaf (magenta line above the graph) and animals with intact cochleae vs. acutely deafened cochleae (cyan line above the graph) using the two-tailed Wilcoxon rank-sum test (false discovery rate corrected, $p < 0.001$). A1 primary auditory cortex, PAF posterior auditory field, LFP local field potentials.
